# Supplementary material for: Altered Expression of NK Receptors in Racially/Ethnically Diverse and Risk-of-Relapse Pediatric Acute Lymphoblastic Leukemia Patients
Source: Biomedicines. 2025 Jun 9;13(6):1412. doi: 10.3390/biomedicines13061412 (PMC12189698; doi:10.3390/biomedicines13061412)
Supplement: Supplementary file 1 [file biomedicines-13-01412-s001.zip › biomedicines-3648405-supplementary.pdf]

Supplementary Table S1. Clinical and demographic characteristics of pediatric patients with ALL

ID	Age/ Sex	Race/ Ethn.	ALL subtype	CNS Status	MRD	DNA Index	Hyper-diploidy	Hypo-diploidy	Tel/AML	Ph+
1001	18/M	H	B cell	1	No	1	No	No	No	No
1002	14/F	C	T cell	2b	Unk	1	No	No	No	No
1003	5/F	H	B cell	1	Yes	1.14	No	No	No	No
1004	4/M	H	B cell	2a	No	1.36	Yes	No	No	No
1005	10/F	AA	T cell	3a	Yes	1.16	Yes	No	No	No
1006	17/M	C	B cell	1	No	1.22	Yes	No	No	No
1007	9/F	H	B cell	1	No	Unk	Unk	Unk	Yes	No
1008	13/F	C	B cell	1	No	Unk	Unk	Unk	No	No
1009	5/M	C	B cell	1	No	Unk	Unk	Unk	No	No
1010	7/M	C	T cell	1	No	1	No	No	No	No
1011	16/M	C	B cell	1	No	1.21	Yes	No	No	No
1012	10/F	AA	B cell	1	No	Unk	Unk	Unk	No	No
1013	12/F	C	B cell	1	No	Unk	Unk	Unk	No	No
1014	3/F	C	B cell	3a	No	1	No	No	No	Yes
1015	15/M	C	B cell	1	No	1.09	No	No	No	No
1016	3/F	C	B cell	1	No	1	No	No	No	No
1017	3/F	H	B cell	2a	No	1.18	Yes	No	No	No
1018	8/F	C	B cell	1	Yes	1.17	Yes	No	No	No
1019	12/F	H	B cell	2a	No	1	No	No	No	Yes
1020	5/F	C	B cell	2a	No	1.20	Yes	No	No	No
1021	12/M	C	B cell	1	Yes	1	No	No	No	No
1024	3/F	Mix	B cell	2a	No	Unk	Unk	Unk	No	No
1025	11/M	C	B cell	1	No	1	No	No	No	No
1026	2/F	H	B cell	1	Yes	1	No	No	No	No
1027	8/F	H	B cell	1	No	1	No	No	No	No
1028	19/F	C	B cell	1	No	Unk	Yes	No	No	No
1030	5/M	C	B cell	1	No	1.13	No	No	No	No
1032	4/M	H	B cell	1	No	Unk	Unk	Yes	Yes	No
1033	12/M	H	B cell	1	No	Unk	Unk	Unk	No	Yes
1034	2/M	C	B cell	1	No	Unk	Unk	Unk	No	No
1035	10/F	C	B cell	1	No	Unk	Unk	Unk	No	Yes
1036	10/M	C	B cell	2a	No	Unk	Unk	Unk	No	No
1037	13/M	H	B cell	1	No	1	No	No	No	No
1038	5/M	H	B cell	1	No	9.99	No	No	No	No
1039	3/M	C	B cell	1	Yes	9.99	Yes	No	No	No
1040	5/F	C	B cell	1	No	9.99	No	No	No	No
1041	5/F	C	B cell	1	No	9.99	No	No	No	No
1042	5/M	C	B cell	1	Unk	9.99	No	No	No	Yes
1043	8/M	H	B cell	2c	No	9.99	Yes	No	No	No
1044	20/M	C	B cell	2b	No	9.99	Yes	No	No	Yes
1045	13/F	H	B cell	2c	No	9.99	No	No	No	No
1046	2/M	C	B cell	1	No	9.99	Yes	No	No	No

AA = African American; C = Caucasian; H = Hispanic; Mix = mixed race/ethnicity; M = male; F = female; Unk = Unknown; Ph+ = Philadelphia chromosome positive

Supplementary Figure

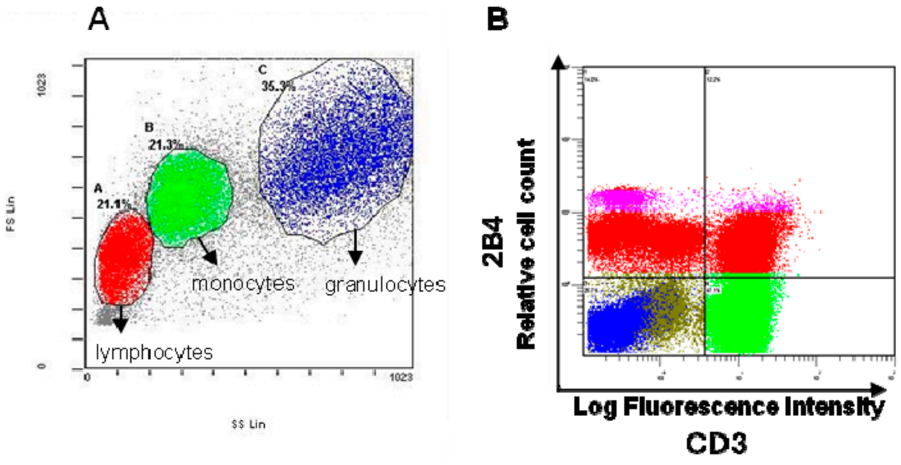

Figure S1: Gating strategy for flow cytometry. (A) Immune cell populations were gated based on forward and side scatter to distinguish lymphocytes and monocytes from other cells according to size and granularity. (B) Representative dot plots from a patient sample shows CD3+ 2B4+ cells in the upper right quadrant.
